# Managing sustainable practices and logistics value to improve customer loyalty: importers vs. freight forwarders

**DOI:** 10.1007/s13437-023-00299-1

**Published:** 2023-01-23

**Authors:** Milva Eileen Justavino-Castillo, Irene Gil-Saura, Maria Fuentes-Blasco, Beatriz Moliner-Velázquez, David Servera-Francés

**Affiliations:** 1 Department of Logistics, Technological University of Panama, Avenida Ricardo J. Alfaro, Campus Metropolitano Dr. Víctor Levi Sasso, Panamá, Panama; 2 Marketing and Market Research Department, Economy Faculty, University of Valencia, Avda. Tarongers, S/N, 46022 Valencia, Spain; 3 Business Management and Marketing Department, Faculty of Business, Pablo de Olavide University, Ctra. de Utrera, Km. 1, 41013 Seville, Spain; 4 Marketing and Market Research Department, Economy Faculty, University of Valencia, Avda. Tarongers, S/N, 46022 Valencia, Spain; 5 Economy, Management and Marketing Department, Faculty of Economy and Business, Catholic University of Valencia, C/Corona 34, 46003 Valencia, Spain

**Keywords:** Sustainable practices, Logistics value, Loyalty, Importers, Maritime freight transport, Panama

## Abstract

In B2B context, this study focuses on analysing the loyalty formation process from a set of antecedent multidimensional variables—sustainable practices and logistics value—and the moderating effect of type of customer in maritime transport from the user’s perspective. Based on a sample of 105 importing companies and freight forwarders in Panama, a structural equation model was estimated to test the research hypotheses. The results show the significant influences of sustainable practices on value and value on loyalty. The significant mediating effect of value in the relationship between sustainable practices and loyalty is also verified. It indicates the benefits of the presence of perceived logistics value in B2B relationships since it reinforces the intensity of the links between sustainable practices and loyalty. The moderating role of the type of business was confirmed. Amongst importers, sustainable practices show a significantly greater impact on logistics value than in the freight forwarders group.

## Introduction

The spread of the coronavirus (COVID-19) brought into sharp relief the role that the maritime industry plays as a key facilitator of international trade (UNCTAD 2020). According to UNCTAD (2021), maritime transport is responsible for moving 80% of the world’s cargo. This sector is undergoing a period of profound change due to commercial losses in the supply chain as a result of the international health crisis. Added to this are the pressures exerted by stakeholders for greater sustainability (Alamoush et al. 2021), and rising competitiveness due to perceived commodification in the market (Balci et al. 2018, 2019). In this context, there is a consensus in the literature that shipping companies that wish to remain profitable must develop customer-focused strategies (Balci et al. 2019; Tepe and Arabelen 2022; Yuen et al. 2018b).

Faced with a complex and dynamic market such as maritime transport, relationship marketing has become a disciplinary framework for the development of profitable long-term relationships (Tepe and Arabelen 2022). Along these lines, the literature begins to point out that it is possible to achieve customer loyalty through the implementation of sustainable practices (Shin et al. 2017; Yang 2017; Yuen et al. 2018b; Jozef et al. 2019).

Sustainability is defined from the perspective of the triple bottom line (TBL) that comprises three pillars of support: economic, environmental and social (Elkington 2004). Despite the pressure exerted by stakeholders to comply with sustainability demands based on these three pillars (Yuen et al. 2017), few studies have analysed customer perceptions of these practices, from a TBL approach. In addition, most studies have focused solely on environmental sustainability (Vural et al. 2021), ignoring the interconnection that exists between the three dimensions.

In the business-to-business (B2B) context, some authors agree that environmental aspects influence the evaluation of providers (Yuen et al. 2016a; Tran et al. 2020) . This evaluation is explained through the perceived value. Value is defined as meeting customer logistics expectations in terms of costs and benefits (Gil-Saura et al. 2010, 2018). Given this scenario, the potential of logistics to improve service has been recognised and given the name of “logistics value” (Gil-Saura et al. 2010). In this sense, customers are increasingly sensitive to environmental and social aspects, which leads them to incorporate sustainability requirements into their purchasing decision processes. Some authors have identified this process as socioeconomic sustainability (Spychalska-Wojtkiewicz 2020), which refers to the inclusion of social and environmental aspects in the value proposition made by companies. Sustainability has become a very important requirement for customers, especially for the so-called aware customers, i.e. those customers who consciously make their decisions in line with the UN’s SDGs, or sustainable development goals (Ostrowski 2021). There is no specific SDG for maritime transport, but there are those on which it has a great influence, such as SDG 3: health and wellbeing, and SDG 13: climate action. In this sense, we can identify various sustainable initiatives that can help improve the logistics value offered by the company. For example, carrying out commercial transactions electronically, through the implementation of logistics ICTs such as EDI, helps to reduce paper consumption, becoming an environmentally friendly practice that creates value for the customer by reducing time and costs (Heilig et al. 2017). Another example is the acquisition of larger vessels that contribute to economies of scale. By transporting a large amount of cargo in a single trip, value is created for the customer by obtaining lower freight rates, which reduces logistical cost. In addition, there is a reduction in carbon and sulphur emissions (Benamara et al. 2019; Garg and Kashav 2019). Maritime transport of both cargo and passengers emits carbon dioxide and other gases that cause global warming. Through operational changes and the use of renewable energy, carbon dioxide emissions can be reduced, which improves the climate and reduces logistics costs (Benamara et al. 2019; Garg and Kashav 2019). All these initiatives will contribute to improving the health and wellbeing of employees and the community, as well as reducing the negative impact on the climate. In addition, these sustainability practices reduce operating costs and this is subsequently reflected in the price (Garg and Kashav 2019; Tran et al. 2020). Even more so in the current situation in which maritime transport freight rates have increased more than fivefold compared to 2019 due to the pandemic, and a further increase is expected in 2022 due to the closure of Chinese ports in March (UNCTAD 2022).

In this context of increasing costs and, therefore, the price of freight, the development by shipping companies of strategies aimed at increasing customer loyalty is even more necessary, in order to guarantee their long-term survival. In this sense, previous studies have indicated that a customer will be loyal to their service provider if they perceive that the value of the service received is higher than that of the competition (Hänninen and Karjaluoto 2017; Ruiz-Martínez et al. 2019; Lin et al. 2021).

However, despite the competitive advantages that sustainable practices represent and the logistics value for maritime transport companies, the studies that have analysed their chain reaction, in pursuit of the generation of loyalty, are scarce, and to our knowledge none have come close to its analysis from a multidimensional approach.

With the objective of addressing this gap, our work proposes an advance in the research that will be tested in customers of maritime transport companies. Shipping company customers can be classified into two groups: freight forwarders and shippers. Freight forwarders act as intermediaries between the shipping company and the owner of the cargo. Shippers, both importers and exporters, are the owners of the cargo. They can contract the transport service directly with the shipping company or subcontract it through the forwarder.

Previous studies have explored the attitudes of shippers and freight forwarders towards environmental sustainability. Results have indicated that customer groups have different behaviours regarding environmental activities (Van-den-Berg and De Langen 2017). In the field of research, most of the studies do not classify shippers, therefore, in an attempt to advance knowledge in this field, this study proposes to analyse importers, as part of the group of shippers, and freight forwarders.

Focusing on the customer-service provider relationship in the maritime transport sector, the objectives of this study are as follows. Firstly, to assess the influence of sustainable practices in the logistics value and loyalty chain of effects. Secondly, it is proposed to analyse whether logistics value can play this mediating role. Finally, the role played by the types of customers (importers and freight forwarders) in regard to the proposed relationships, sustainable practices and value, as well as the relationship, logistics value and loyalty, is analysed. The study of these variables will provide a better understanding of the process that leads to the generation of loyalty. This will allow shipping companies to focus their efforts on those aspects that help maintain lasting relationships.

This study is structured as follows. First, there is an overview of the literature related to sustainable practices, logistics value and loyalty. Second, based on the literature review, four hypotheses are developed based on the chain of relationships between the study variables. Third, there is a description of the methodology used followed by the results of the research. Finally, the conclusions and future lines of research are presented.

## Theoretical review

### Sustainable practices in maritime transport

The rapid growth of the maritime sector and the nature of its operations cause a wide-reaching and considerable impact on the environment. This environmental degradation has attracted the attention of stakeholders, who have also expressed concern about other aspects of sustainability (Tran et al. 2020). In maritime transport, sustainability refers to the efforts made by organisations to meet the needs of the present without compromising the ability of future generations to meet their own needs (Yuen et al. 2017).

In this sense, companies can opt for different models of sustainable management. Amongst which stands out corporate social responsibility (CSR), enviromental, social and governance (ESG) criteria, and (TBL). All of them share common elements, such as stakeholder satisfaction, the improvement of society and the reduction of environmental impact. In our work, we have opted for the TBL model since, as García (2015:65) indicates, “TBL is characterised by the establishment of parameters that allow for the assigning of quantitative values to not only economic, but also social and environmental actions of an organisation, using for this the triple result matrix and the establishment of certain standard indicators that offer a degree of objectivity”.

According to Elkington (2004), shipping companies must adopt a strategic position, that is, carry out sustainable activities that contemplate simultaneous improvements in social, economic and environmental performance (Lun et al. 2016). According to Tran et al. (2020), a practice cannot be labelled as sustainable if it has a negative impact on economic performance, regardless of the positive effects in the social and environmental spheres.

In addition, according to institutional theory, shipping companies can receive coercive, mimetic, and regulatory pressures from various stakeholders (Vejvar et al. 2018; Tran et al. 2020). Satisfying the requirements of shareholders implies that shipping companies must carry out practices focused on improving economic performance (Parviainen et al. 2018), for freight forwarders it means seeking synergies in operations that allow for the reduction of operating costs (Ashrafi et al. 2020), for shippers it includes improving social and environmental (Yuen et al. 2016a) and economic (Shin et al. 2017; Van-den-Berg and de Langen 2017) performance, for employees it includes receiving health and safety in the workplace training (Pang and Lu 2018). The consequences derived from non-compliance with these requirements will be extended to the different partners in the chain. It will be the users of the transport services (shippers and freight forwarders) who will be answerable to their customers in the case of any breach of sustainable standards by the shipping company (Jozef et al. 2019), putting their reputation and the loyalty of their customers at risk (Parviainen et al. 2018).

In such a competitive sector, the dominant position of the stakeholders is also explained from the resource dependence theory. Shipping companies are forced to carry out sustainable practices to ensure access to the resources they need to fulfil their operations. For this reason, beyond the pressures exerted by the parties in the chain, the management of sustainable practices is also seen as a reflection of the conduct of the shipping company’s social and environmental activities (Yuen et al. 2017). This behaviour creates links with the different stakeholders, generating functional, social and emotional values that produce customer satisfaction and loyalty (Yuen et al. 2016b).

Through the sustainable development goals (SDGs), the UN emphasises the importance of analysing sustainability from a comprehensive approach (economic, social and environmental), which has produced profound changes in the perspective of CSR (Fasoulis et al. 2019; Gupta and Singh 2020). Currently, CSR is seen as an issue that goes beyond legal compliance, and is considered synonymous with sustainability from its multidimensional approach (Fasoulis and Kurt 2019; Fasoulis et al. 2019).

The various activities and sustainable practices based on the TBL approach, i.e. practices in the social, economic and environmental spheres, are detailed below.

#### Economic practices

The economic performance of shipping companies is strengthened through sustainable practices because they improve resource efficiency (Vural et al. 2021). In other words, profits can be maximised through activities such as speed reduction and the use of more efficient ship engines, since through these practices and investment in this type of technology, fuel consumption is lower (Lam 2015; Yuen et al. 2018a). The availability of economic resources also allows accessibility to new markets, greater infrastructure capacity and improved competitiveness (Psaraftis 2019).

#### Social practices

The social pillar of the TBL focuses on human factors, that is, it considers the needs of employees and the community (Fernando et al. 2019). This dimension is concerned with cultural preservation, the wellbeing of society, health and occupational safety (Psaraftis 2019). Participating in volunteer activities or cooperating with education are social aspects that shipping companies should practice (Yuen et al. 2018a). According to Tran et al. (2020), shipping companies must take an altruistic approach in carrying out their activities.

#### Environmental practices

Environmental practices seek to reduce greenhouse gases, ballast water, hazardous materials, and accidents that affect the environment (Fernando et al. 2019; Psaraftis 2019). Shipping companies respond to these environmental challenges through the acquisition of environmental-friendly ships (Tran et al. 2020) and the use of low-carbon alternative energy sources (Ren and Lützen 2017). This dimension, apart from the transport service, also includes activities related to documentation and shipping materials aimed at reducing the use of materials generated during these operations (Jozef et al. 2019).

### Logistics value

From a relational perspective, creating value for a customer is necessary for the continuity of the relationship between provider and client (Hänninen and Karjaluoto 2017). Therefore, in the logistics field, analysing value has become a fundamental part of gaining competitive advantages. According to Novack et al. (1995) , when a logistics service is offered that meets the customer’s requirements, in terms of benefits and costs, and the customer perceives it that way, logistics value is achieved. Gil-Saura et al. (2010) analysed the antecedents and the results of the logistics value from the perceived value. Their results indicated that logistics value contributes to the generation of the classic satisfaction-loyalty chain. Along these same lines, Kim and Kim (2019) and Lin et al. (2021) analysed the value in maritime transport and indicated that shipping companies must link their customers’ service preferences with the fulfilment of their perception of value. For Nguyen et al. (2019), supplier companies should be concerned with analysing, creating and delivering value to client companies. In other words, shipping companies must focus their resources on offering a high-value service, increasing the benefits perceived by customers and reducing the costs or sacrifices associated with the service process. This premise is also supported by social exchange theory, which states that a company's intentions to continue using a service are based on perceived rewards and transaction costs (Balci et al. 2019).

The activities of the maritime sector go beyond the transport of goods since it covers other aspects such as unloading, storage and information (Kim and Kim 2019). According to Yazdanparast et al. (2010), these activities will generate logistics value if they are managed efficiently and effectively, and the client perceives it as such. Along these lines, previous studies have indicated that value is formed from tangible and intangible variables. Kim and Kim (2020) indicate that price and service experience are determining factors in perceived value. Other studies pointed to the importance of time (Jozef et al. 2019) and quality as factors influencing the decision to choose a shipping company (Yuen and Thai 2015).

Therefore, the perceived value, from a trade-off approach, is a determining variable in customers’ purchasing decisions. However, the literature has paid limited attention to the study of perceived value in transportation. In this context, this research adopts, as a conceptual framework, the study by Servera-Francés et al. (2008) and Gil-Saura et al. (2010), since they have used a research scenario very similar to this one. Both investigations analyse the generation of value in a B2B environment, within the service sector. In addition, they focus on analysing the perceptions of client companies regarding the service received from their provider. On the other hand, the selected client companies have a logistics department; therefore, the evaluation of the service is carried out from a logistics approach.

Under this panorama, and taking into account the complexity of the sector, this study understands logistics value as a multidimensional construct. Based on the adaptation by Gil-Saura et al. (2010) to the study by Novack et al. (1995), logistics value is conceptualised in terms of productivity of the logistic services, the importance of the logistic services and the quantification of the value. These three factors are perfectly suited to shipping. Since, as we have commented in the introduction, the costs of maritime transport are increasing significantly, which makes the productivity of the logistics service essential as a measure to increase the logistics value (the higher the productivity, the lower the costs). On the other hand, the high competitiveness and fragmentation of maritime transport also stands out, which means that companies that want to generate value will do so through the generation of competitive advantages linked to the improvement of the logistics service (importance factor of the logistics service). Finally, the logistics value will be greater in those companies that quantify the same position as they will be more aware of the effectiveness of the measures taken (Servera-Francés et al. 2008) .

#### Quantification of the logistics value

There is a consensus in the literature that price is a dominant factor when selecting a shipping company. According to Lin et al. (2021), shipping company customers pay for the perceived value of the service received. This indicates that in order to generate logistics value, service provider companies must work to minimise the costs of the service offered and this reduction must be reflected in the price. For Yuen et al. (2018b) , through the transaction costs, it is possible to reduce the total cost of the service. Automated reservation requests, return of confirmations and invoice issuance are practices that could generate value by reducing the aforementioned transaction costs (Jozef et al. 2019).

#### Productivity of the logistics service

In the study of the cost–benefit relationship generated in the provision of services, productivity plays a very important role. Service productivity refers to the way in which a company manages its resources in order to meet the objectives set (Dobmeier 2016). Shipping companies have physical, human, capital and technological resources. The way these resources are combined and allocated reflects the operational quality of the business (Linh et al. 2019). According to Grönroos and Ojasalo (2004), service productivity must also be evaluated from the customer’s perspective.

#### Importance of the logistics service

In recent years, shipping company customers have become more price sensitive, as they find shipping company services to be standardised due to strategic ship-sharing alliances (Balci et al. 2018; 2019). This implies that shipping companies must offer services that are seen to be of higher value compared to the services of the competition (Balci et al. 2019). If a customer receives a higher value service, there is a reduced likelihood that they will opt for another carrier. Since maritime transport is an intangible service, Balci et al. (2019) indicated that through the social exchange theory approach and relational linkage strategies (financial, social, and structural), it is possible to increase the benefits perceived by customers and thus contribute to the generation of loyalty.

### Loyalty

Loyalty is defined as the intention of client companies to continue the relationship with their shipping service provider (Jozef et al. 2019). The conceptualisation of loyalty has been carried out from an attitudinal and behavioural approach (Colmenares and Saavedra 2007), which asserts that it is generated from the gradual accumulation of positive encounters between the provider and the customer (Oliver 1999). For Dike and Stanley (2021), the search for customer loyalty is more frequent in service customers than in customers of tangible products, and is of paramount importance in the context of services when explaining the links between providers and customers. Loyalty towards the main service provider materialises through the repurchase and recommendation of the shipping company to other companies (Shin et al. 2017). In this way, building long-term relationships based on achieving loyalty is seen as a form of protection against price competition (Balci et al. 2019).

## Model proposal and hypothesis

### Effect of sustainable practices and logistics value

The perceived value is produced from the benefits that the clients perceive due to the shipping companies’ participation in sustainable activities. In the field of marketing, there are various works that link the application of sustainable practices with the increase in perceived value (Luo and Bhattacharya 2006; Servera-Francés et al. 2020) .

Regarding maritime transport, the literature indicates that the participation of shipping companies in the development of sustainable practices generates value (Yuen et al. 2018b). In line with resource-based theory, sustainability is defined as a set of capabilities that allow resources to be continuously deployed, combined, reconfigured and integrated in a dynamic manner (Yuen et al. 2019). This agility in the use of resources is perceived by the client as the commitment that shipping companies have with sustainability (Tran et al. 2020). In contrast, previous studies have highlighted that customers tend to prioritize economic factors in their purchasing decisions (Van-den-Berg and de Langen 2015). However, investing in sustainability also allows makes it possible to create an economic profit. For example, fuel consumption represents 60% of the operating costs of ships. The use of ships with more efficient engines will reduce this consumption and this reduction will be reflected in the general costs of the trip and therefore in the final price paid by the customer (Seddiek and Ammar 2020) . On the other hand, according to contingency theory, sustainable activities drive differentiation through the incorporation of technologies and new services, e.g. mobile applications (Tran et al. 2020; Yuen et al. 2019). Furthermore, sustainability has the property of being measurable (Vural et al. 2021; Zhou et al. 2021). Previous studies have indicated that sustainability reporting is valued by stakeholders, who use it as a tool to award bids. Therefore, these reports should be made up of indicators that allow stakeholders to reduce uncertainty about the sustainable activities practised by shipping companies (Zhou et al. 2021). Along these lines, Arslanagic-Kalajdzic and Zabkar (2017) indicate that the activities carried out as CSR are perceived as signs of the quality of the company's service, which generates value for the customer.

Therefore, based on the above, the first hypothesis is proposed:

- H_1_: Sustainable practices have a direct and positive effect on logistics value.

### Effect of logistics value and loyalty

Social exchange theory argues that firms continue or end a relationship by evaluating transaction costs and rewards. Balci et al. (2019) applied this theory to the shipping company-customer relationship and concluded that social, structural and economic bonding strategies strengthen relationships with customers. Lin et al. (2021), in the context of short sea shipping, concluded that there is a strong link between the value perceived by customers and their purchase intentions. For their part, the studies by Yuen et al. (2018b) demonstrated the importance of providing services that are perceived by customers as superior, compared to other shipping companies, since this will lead to customer loyalty. Therefore, our next hypothesis states that:

- H_2_: Logistics value has a direct and positive effect on loyalty.

### Mediating effect of logistics value

Previous studies have indicated the indirect relationship of sustainable activities with loyalty (He and Lai 2014; Yuen et al. 2016b, 2018a). Yuen et al. (2016b) analysed corporate social responsibility (social and environmental aspects) with the customer’s willingness to pay more for CSR. It is understood that if a customer pays more for CSR, it is because they accept the price increase to continue their commitment with their supplier. They concluded that this link is mediated by personal beliefs and perceived favourability. Yuen et al. (2018a) analysed the role of perceived value in the customer–supplier company relationship; the results indicated that sustainable shipping practices are a necessary but insufficient condition for earning the loyalty of the shipping company’s customers. Therefore, they emphasise that sustainable practices must create value and be perceived as such by customers in order to gain their loyalty. In addition, the performance of sustainable activities contributes to the environmental image of companies, which is a factor that adds to the customers’ perceived value. For their part, Jozef et al. (2019) pointed out that shippers increase their loyalty levels when they perceive that their service provider implements sustainable practices. Despite the value contributions, in the relationship between sustainable practices and loyalty, there are few studies that have analysed value as a mediating variable in the B2B sector. However, there is evidence of the mediating effect of value in the literature on consumer behaviour (B2C). Based on the belief that it is possible to transfer findings between B2C and B2B contexts (Gil-Saura et al 2020), we moved forward with the study of the mediating role of logistics value, and its potential role as a driver of loyalty. Therefore, based on the positive effects generated by the participation of companies in social activities (Hanaysha 2018; Mohammed and Al-Swidi 2019), we propose the following hypothesis:

- H_3_: Logistics value mediates the relationship between sustainable practices and loyalty.

### Moderating effect of the customer type variable

Although the shipping service purchasing process of freight forwarders and shippers are similar, the position they have within the maritime supply chain is different; therefore, their service provider selection criteria vary (Feo-Valero and Martínez-Moya 2022). In the case of freight forwarders, by taking on the role of intermediaries, their choices are based on the demands of their customers (Jozef et al. 2019) who demand a diversity of services depending on the type of industry to which they belong. Feo-Valero and Martínez-Moya (2022) indicated that freight forwarders are more sensitive to price than shippers because they must obtain a profit margin. In addition, they concluded that shippers tend to change service providers more frequently than freight forwarders.

Previous studies have analysed the perceptions of shipping company clients regarding sustainability (Van-den-Berg and De Langen 2017) and logistics value (Lin et al. 2021). These investigations have shown that in the transport sector, perceptions differ depending on the customer group. According to Van-den-Berg and De Langen (2017), freight forwarders are less concerned about environmental sustainability than shippers. Gil-Saura et al. (2015) identified in the transport sector groups of companies differentiated by the perceived level of logistics value.

These investigations have shown that in the transport sector, perceptions differ depending on the customer group.

In order to obtain a more accurate picture of the position that these two groups of clients have in terms of their perceptions of sustainable practices and logistics value, importing and freight forwarding clients are included as a moderator variable. In this way, it will be possible to know if, depending on the type of customer, the proposed relationships of sustainability and logistics value and the relationship of logistics value and loyalty change.

For this study, only importers have been considered within the group of shippers, excluding exporters. The reason for choosing importers is because imports in Panama exceed exports by more than 50%. In such a way that we understand that the greater the volume of business, the greater the knowledge the importing companies will have about maritime transport. This is a more valid interlocutor for the proposed study.

Therefore, we consider that the customer company type variable can act as a moderator in the relationships that we describe below:

- H_4a_: Customer company types (importers and freight forwarders) moderate the relationship between sustainable practices and logistics value.
- H_4b_. Customer company types (importers and freight forwarders) moderate the relationship between logistics value and loyalty.

**Fig. 1:**
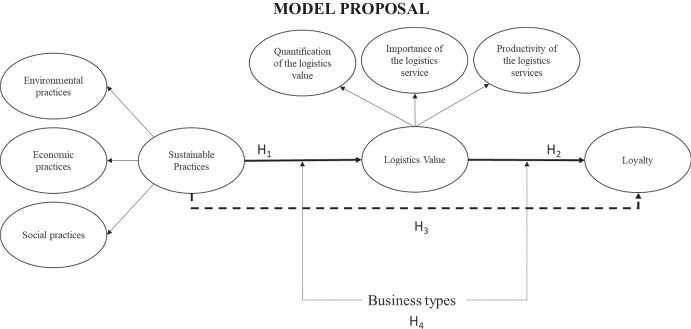
Model proposal

The hypotheses proposed are shown in Fig. 1.

To control potential biases in the relationships estimations, seniority in the sector and company size have been added as control variables. Seniority in the sector refers to the number of years that the client company has operated in the sector (Hirvonen et al. 2016). Over time, companies gain an understanding of the market. In addition, stakeholders are aware of their existence and therefore higher commercial expectations are formed. This experience and market recognition can create a closer relationship with the service provider through incentives such as special freight rates (Hirvonen et al. 2016; Yuen et al. 2018a). The size of the company refers to the number of workers (Hirvonen et al. 2016). We consider it appropriate to use these control variables, seniority in the sector and company size, in order to rule out that loyalty ties are generated from incentives that client companies obtain due to their size and time in the market.

## Materials and methods

### Measurement instrument and field work

Empirical quantitative research applied to maritime transport services was developed through a structured questionnaire, targeted at the individuals responsible for hiring the shipping company for the maritime transport service. Both importers and freight forwarders at a managerial level of the Republic of Panama were included in the sample. The choice of this geographical area is due to the important transformations that its maritime business is undergoing, caused by, amongst other factors, the growing economic and political power of China, the long-term impacts of COVID-19, climate change, and technology trends (CSIS 2021). The companies under study are active in the channel, which operates under a continuous work scheme 24 h a day, every day of the year. According to the latest data available, in 2020, 255,733,585 tonnes of cargo were moved, which represents an increase of 1.32% compared to the previous year, and is the highest figure since 2010 (Meem 2021). In addition, the wide range of goods transported through this channel is remarkable compared to other routes, from basic products and consumables, to high-tech and manufactured goods (Georgia Tech Panama Innovation and Research Center 2022).

The measurement scale developed by Shin et al. (2017) was used to evaluate sustainable practices. The multidimensional nature of sustainable practices was considered, taking into account three dimensions: economic, social and environmental sustainability. The scale of Servera-Francés et al. (2008) was used to measure the logistics value. Following the proposal of these authors, the logistics value is measured through the dimensions of productivity of the logistics service, importance of the logistics service and quantification of the logistics service. Finally, to assess loyalty, a unidimensional measure of 5 items was considered based on Shin et al. (2017). In all cases, 7-point Likert scales were used (from 1 “strongly disagree” to 7 “strongly agree”) in which the respondent had to position themself according to their degree of agreement with the proposed statements (see Appendix I).

The final sample was delimited based on secondary information from LEGISCOMEX, Ministry of Commerce and Industries (MICI), Colón Free Trade Zone, Association of Cargo Handling Agencies in Panama (APAC). The field work was carried out between the months of October 2019 and February 2020. One hundred five valid and complete responses were obtained. The main characteristics of the sample are shown in Table 1.

**Table 1:** Sample profile

Activity	Number	%	Number of employees	Number	%
Importer	31	29.52	1–25	51	48.57
Freight forwarder	74	70.48	26–50	31	29.52
**Time in the maritime sector**			51–100	12	11.43
1–5 years	27	25.71	> 101	10	9.53
6–10 years	32	30.48	Companies which did not reply	1	0.95
11–15 years	16	15.25	**Length of patronage with the main shipping company**		
16–20 years	7	6.66	< 1 year	3	2.86
> 20 years	18	17.14	1–4 years	43	40.95
Companies which did not reply	5	4.76	5–9 years	38	36.19
			≥ 10 years	19	18.10
			Non response	2	1.90

### Data analysis

The methodology used for the hypothesis contrast was the estimation of a structural equation model through partial least squares (PLS) path modelling. Taking into consideration previous studies, the modelling of sustainable practices and logistics value was proposed as a reflective-reflective model. The study by Khan et al. (2016) indicated that reflective-type sustainability factors are appropriate in the supply chain and micro-enterprises. Kumar and Goswami (2019) validated the dimensions of sustainability in both the service and manufacturing industries. They indicated that the measurement elements of sustainable practices, first-order constructs, are characterised by being interchangeable and having a high correlation. Regarding the logistics value, the contributions of Servera-Francés et al. (2008) and Gil-Saura et al. (2010) have validated the reflective character of this variable through their studies carried out in companies dedicated to various commercial activities. In the same vein, Justavino-Castillo et al. (2020) applied reflective measures to analyse logistics value in the maritime sector, confirming the existence of a causal relationship between the logistics value variable and the indicators.

Seniority in the sector and company size were included as control variables in the modelling.

In the first step, the loads of the indicators were examined, estimating the second-order measurement model (see Table 2) based on the factorial scores obtained from the dimensions of sustainable practices and logistics value. According to Hair et al. (2019), an item is significant if its loadings are greater than 0.708, which means that the factor explains 50% of its variance.

**Table 2:** Second-order measurement model estimation: reliability, consistency and convergent validity

Construct	Indicators	Loading	t-Stat	Cronbach's alpha	Composite reliability	AVE
Sustainable practices	Environmental practices Economic practices Social practices	0.908** 0.923** 0.938**	23.55 41.00 48.75	0.913	0.945	0.852
Logistics value	Quantification of the logistics value Importance of the logistics service Productivity of the logistic service	0.892** 0.957** 0.954**	13.26 42.20 46.90	0.928	0.954	0.874
Loyalty	LO1 LO2 LO3 LO4 LO5	0.914** 0.912** 0.841** 0.881** 0.887**	24.70 27.37 9.70 22.66 33.98	0.932	0.949	0.788

^**^p < 0.01

In the second step, two indicators were used to measure the internal consistency of the dimensions: Cronbach’s alpha and composite reliability (CR). In both cases, the recommended minimum threshold of 0.7 was exceeded (Anderson and Gerbing 1988). In the third step of the evaluation of the first-order reflective measurement model, the convergent validity was analysed through the average variance extracted (AVE), whose value must exceed 0.5 (Hair et al. 2019).

In the fourth step, this study tests the discriminant validity between the constructs using the Fornell and Larcker (1981) criterion and the heterotrait-monotrait ratio (HTMT). It can be affirmed that the discriminant validity is fulfilled given that the square root of the AVE is greater than the correlation between constructs. The discriminant validity is also confirmed by the values of the squared correlations of the HTMT relationship that are less than the value of 0.9 (see Table 3) (Henseler et al. 2015).

**Table 3:** Discriminant validity assessment

	Firm age (control)	Loyalty	Sustainable practices	Firm size (control)	Logistics value
1.Firm age (control)	*–*	0.053	0.083	0.365	0.016
2. Loyalty	− 0.025	*0.887*	0.745	0.032	0.807
3. Sustainable practices	0.017	0.689	*0.923*	0.078	0.797
4. Firm size (control)	0.365	0.007	− 0.073	*–*	0.037
5. Logistics Value	− 0.01	0.757	0.741	− 0.025	*0.935*

The diagonal elements (in italics) are the square root of the AVE. Values below the diagonal elements are the inter-construct correlations. Values above the diagonal indicate the HTMT ratio

## Results

### Structural model

As suggested by Hair et al. (2019), the R^2^ values, standardised β values and *t* values were evaluated using a nonparametric bootstrapping procedure with 5000 subsamples and *Q*^2^ predictive relevance to evaluate the structural model. R^2^ values were above the minimum threshold of 0.1 (Falk and Miller 1992) and *Q*^2^ values were greater than 0, confirming the predictive relevance of the model. This made it possible to assess the significance of previously established causal relationships (Chin 1998).

The results of the estimates support the existence of a direct and significant effect in the relationship between sustainable practices and logistics value, in support of H_1_. This result is consistent with previous research at the B2C level (Currás-Pérez et al. 2018; Servera-Francés et al. 2020), and at the B2B level (Arslanagic-Kalajdzic and Zabkar 2017; Yuen et al. 2018b). In this way, it is demonstrated that the various dimensions of sustainability generate different benefits that are perceived by the client. According to Yuen et al. (2018b) , the benefits generated by sustainable practices can be economic, quality, emotional and socially useful. This result emphasises the importance of companies communicating sustainable activities to create value in the client company (Arslanagic-Kalajdzic and Zabkar 2017).

H_2_ is also accepted, as the results verify that logistics value has a direct and significant effect on loyalty. In line with previous literature (Hänninen and Karjaluoto 2017; Lin et al. 2021; Ruiz-Martínez et al. 2019; Yuan et al. 2020), this study underlines the importance of business relationship management. In any B2B relationship, there must be mutual benefits. Customers seek superior value and providers seek customer loyalty (Hänninen and Karjaluoto 2017). Therefore, shipping companies must offer superior value to the competition to maintain loyal customers.

Regarding H_3_, the indirect effect on the relationship between sustainable practices and loyalty turned out to be significant, thereby confirming the mediating role of the logistics value variable. The R^2^ value for loyalty indicates that 61.3% of the variation in loyalty amongst shipping company customers can be explained through logistics value and sustainable practices. The results are presented in Table 4. This finding is in line with the work of Yuen et al. (2018b) , who verified the mediating effect of perceived value in maritime transport. In addition, previous studies such as that of Shin et al. (2017) indicated that the effect of sustainability on loyalty is mediated by satisfaction. In conclusion, sustainable activities have an indirect effect on customer loyalty (Yuen et al. 2018b). Customers expect to perceive value in the services that are provided in the maritime sector (Garg and Kashav 2019). This indicates that service providers must take an integrative approach of resources to create value. For this, greater interaction and collaboration between the actors of the maritime sector is necessary: shipper, freight forwarder, shipping company (Vural et al. 2019). This interaction will also allow innovative proposals to be developed in the maritime sector, along with technological aspects (Makkonen and Inkinen 2021) , which will contribute to the creation of value.

**Table 4:** Structural model (direct and indirect effects)

**Direct effect**	β (t-Stat)	f^2^
Sustainable practices → Logistics Value	0.741**(7.77)	1.216
Logistics value → Loyalty	0.541**(4.23)	0.340
Sustainable practices → Loyalty	0.293** (2.62)	0.099
*Firm age *→* Loyalty*	− *0.046 (0.60)*	
*Firm size *→* Loyalty*	*0.059 (0.64)*	
**Indirect effect**		
Sustainable practices → loyalty	0.401**(3.33)	

R^2^ (loyalty) = 0.613; R^2^ (logistics value) = 0.549; Q^2^ (loyalty) = 0.447; Q^2^ (logistics value) = 0.516; VAF = 57.78%; **p < 0.01

### Multigroup analysis

Before confirming the possible differences in the relationships depending on business type (importers and freight forwarders), the existence of metric invariance was checked. This was evaluated following the three steps of the MICOM procedure (Henseler et al. 2016) (see Table 5). First, the configuration invariance between the samples was determined since the same items, the same collection algorithm and the same data treatment were used for all the constructs in the two groups. In step 2, the permutation test indicates that none of the c values differ significantly between the samples, since c = 1 is within the lower and upper limits of the 95% confidence intervals, confirming the compound invariance. Finally, in steps 3a-3b, the equality of variances and means between the constructs could not be confirmed, since, in some cases, the difference of the composite scores is outside the limits of the 95% confidence interval. Therefore, complete invariance could not be determined, although partial was evident, as the minimum condition to compare standardised path coefficients between groups (Hair et al. 2017).

**Table 5:** Measurement invariance test (MICOM)

**Variable**	**Step 1**	**Step 2**		Partial Measurement invariance	**Step 3a**			**Step 3b**		
	Configural invariance	Compositional invariance			Equal Means Assessment			Equal variances assessment		
		C = 1	5%	Established difference	Difference	Confidence interval	Equal	Difference	Interval	Equal
Firm age	Yes	1	1	Yes	0.271	[− 0.344;0.359]	Yes	0.261	[− 0.445 − 0.342]	Yes
Loyalty	Yes	0.994	0.989	Yes	− 0.615	[− 0.361;0.354]	No	1.513	[− 1.246 − 1.181]	No
Sustainable practices	Yes	1	0.996	Yes	− 0.798	[− 0.381;0.349]	No	1.294	[− 1.007 − 1.001]	No
Firm size	Yes	1	1	Yes	0.485	[− 0.311;0.378]	No	0.783	[− 1.145 − 0.880]	Yes
Logistics value	Yes	0.981	0.984	No	− 0.817	[− 0.383;0.327]	No	2.048	[1.557 − 1.675]	No

Table 6 shows the results of the multigroup analysis evaluation using the nonparametric permutation test. The permutation test is considered one of the most conservative PLS-SEM techniques for evaluating the differences in trajectory coefficients between two groups (Sarstedt et al. 2011).

**Table 6:** Multigroup analysis

Paths	Importers (G1) *N* = 31	Freight forwarders (G2) *N* = 74	G1 vs. G2	*p* value
	β (t-Stat)	β (t-Stat)	|Diff. paths|	
Sustainable practices → Logistics value	0.860** (8.73)	0.500** (5.48)	0.360**	0.009
Logistics value → Loyalty	0.841** (3.93)	0.313* (2.10)	0.528*	0.023
Sustainable practices → Loyalty	0.045 (0.22)	0.357** (2.40)	− 0.313	0.111
*Firm age *→* Loyalty*	0.059 (0.60)	− 0.116 (0.88)	0.176	0.123
*Firm size *→* Loyalty*	− 0.001 (0.00)	0.023 (0.14)	− 0.024	0.472

^*^p < 0.05; **p < 0.01

Based on the permutation results obtained, it is verified that there are significant differences between the importer and freight forwarder customers. In the group of importers, sustainable practices have a significantly greater effect on logistics value. The results also indicate that the relationship between logistics value and loyalty is stronger in the importers group than in the freight forwarders group. Therefore, the fourth hypothesis (H_4_) is accepted by corroborating the role of the type of business as a moderating variable. The inclusion of the type of customer variable as a moderating variable has also provided relevant information on the perceptions that customers have about the relationships studied. This work extends the result obtained by Van-den-Berg and de Langen (2017), who explored the perceptions of freight forwarders and shippers in the environmental dimension of sustainability. However, our study analyses clients’ perceptions of sustainability from an overall TBL approach, through the moderator variable. The results agree that shippers show greater interest in sustainability than freight forwarders.

## Conclusions

The objective of this study has been to understand how sustainable practices from a TBL approach lead to the generation of loyalty of shipping companies towards their supplier through logistics value, and to observe the moderating effect of the business type variable on the chain of relationships. In line with the contribution of Yuen et al. (2018b) , the results confirm that sustainable practices influence logistics value, i.e. customers perceive that sustainable activities contribute to maximising their profits. Evidence has also been found that the perceived logistics value has effects on loyalty, confirming that it is an import factor when seeking to build lasting relationships over time, and making it possible to define strategic objectives linked to achieving customer loyalty. This finding is added to the results of previous research, such as that of Hänninen and Karjaluoto (2017) or Ruiz-Martínez et al. (2019), who have confirmed the influence of value on loyalty, and allows progress in the knowledge that these links also exist in the maritime transport of goods.

In addition, the results achieved also show the key role played by logistics value as a mediating variable, that is, they corroborate the benefits of the presence of perceived logistics value in B2B relationships, to the extent that it reinforces the intensity of the links between sustainable practices and loyalty (Hanaysha 2018; Mohammed and Al-Swidi 2019). On the other hand, the direct effect of sustainable practices on loyalty is confirmed, although these results differ from those obtained by Yuen et al. (2018b) . In light of these findings, it is concluded that there is a need to continue investigating loyalty in the field of relationships between companies, especially when their origins lie in the development of sustainable practices. This area of research undoubtedly requires additional contributions that more clearly trace the route to loyalty.

On the other hand, the control variables did not show significant effects on loyalty. From this result, it is concluded that neither the age nor the size of the company contributes significantly to explaining and predicting loyalty; however, the logistics value does.

Regarding the different perceptions of importers and freight forwarders, as a type of shipping company customer, it is evident that importers are more concerned about aspects related to sustainability. These results are consistent with the study by Van-den-Berg and De Langen (2017) who indicated that shippers focus more on environmental sustainability than freight forwarders. As a consequence, compared to the group of freight forwarders, in the group of importers the effects of sustainable practices are better explained through the perceived logistics value and higher levels of loyalty are generated. In conclusion, the perceptions of sustainability from a TBL approach, in these two types of business, allows us clearer insight into the impact on the formation of loyalty.

The results of this study also have managerial implications. Through logistics management, the shipping industry has great opportunities to generate value by considering the development of sustainable practices as a starting point. In other words, through the development of these activities, a higher level of customer service can be provided. To obtain these results, it is necessary for shipping companies to develop sustainable practices from a multidimensional approach. This involves identifying the activities that customers consider to be a priority in each of the pillars of the triple bottom line, since customers consider to be of equal importance the environmental aspect: for example, that shipping companies comply with international guidelines; the social aspect: for example, the training that shipping companies provide to their personnel; and the economic aspect: since customers appreciate that the activities carried out by shipping companies contribute to the economic growth of society. This will allow shipping companies to make appropriate resource investment decisions. In addition, they must develop strategies where sustainable practices contribute to cultivating logistics value, and that are consistent with customer perceptions regarding the productivity, importance and quantification of the logistics service. Communication about the participation of shipping companies in sustainable activities contributes to their positioning in the market (Vural et al. 2021), so we suggest shipping companies use different sustainability disclosure mechanisms from the TBL approach. This is important because it has been shown that customer perceptions regarding sustainable activities lead to the generation of positive effects, expressed in terms of benefits and gaining customer loyalty (Tran et al. 2020). In addition, shipping companies must strive to meet the sustainability requirements of importers and freight forwarders, since it has been shown that the participation of shipping companies in sustainable activities influences the generation of loyalty.

Finally, regarding the limitations and opportunities for future research, the limited scope of the study can be affirmed, given that the data was only collected in the Republic of Panama. In this sense, it should be replicated in other geographical contexts where the maritime transport of merchandise is an important part of the country's economy. In line with the work by Yuen et al. (2016b), we believe that replicating this study in other countries will also enrich the results by determining whether clients' perceptions of sustainability are influenced by their belief systems, culture and social values, as well as their level of economic development. In addition, due to the scarce evidence in the literature on the mediating role of perceived logistics value in the relationship between sustainable practices and loyalty in the B2B context, it is recommended to replicate the study in another type of service industry. On the other hand, other moderating variables could also be included in the model, such as the size of the company or the number of containers it handles.

## Data Availability

Data will be made available on request. Contact the Corresponding Author.

## Appendix I

**Table:**

Measurement Scales	Total sample			Importers			freight forwarders		t test Diff. of means
	*n* = 105			*n* = 31			*n* = 74		
	Mean	SD	Mean		SD	Mean		SD	
Environmental Practices									
EPA1. My main shipping company reduces CO2 emissions by slow steaming of its fleet	5.15	1.44	4.52		1.43	5.46		1.36	10.21**
EPA2. My main shipping company suitably manages ballast water to protect the oceans from environment pollutions	5.24	1.33	5.58		1.28	5.55		1.24	13.17**
EPA3. My main shipping company duly complies to international standards set up by the International Maritime Organization	5.74	1.19	5.42		1.43	5.86		1.06	3.30
EPA4. My main shipping company pays much attention environment protection	5.55	1.27	4.77		1.49	5.89		0.99	19.96**
EPA5. My company uses environmental-friendly materials and equipment (e.g. nontoxic paint, electric deck machine, ballast water system)	5.36	1.31	4.76		1.38	5.62		1.21	9.65**
EPA6. My company adopts environmental-friendly shipbuilding designs (e.g. improved engine design and waste heat recovery systems)	5.38	1.17	4.83		1.29	5.60		1.03	10.42**
Social Practices									
SP1. My main shipping company encourages cooperation with regional communities and educational institutions	5.36	1.36	4.80		1.54	5.59		1.22	7.82*
SP2. My main shipping company carries out its corporate social responsibility in proportion to its sales	5.54	1.27	4.80		1.45	5.85		1.04	17.17**
SP3. My main shipping company supports additional education for its staffs	5.60	1.29	5.20		1.52	5.78		1.15	4.39*
SP4. My main shipping company encourages its staffs to involve in the voluntary activities in the community	5.31	1.31	4.61		1.56	5.60		1.07	14.19**
SP5. My company donates to charitable organization	5.32	1.27	4.80		1.51	5.54		1.10	7.71*
Economic Practices									
EP1. My main shipping company’s economic activities contribute to the economic growth of the society to which it belongs	5.87	1.16	5.55		1.63	6.01		0.88	3.56
EP2. My main shipping company tries to enlarge employment from the local community	5.66	1.21	5.26		1.61	5.82		0.95	4.98*
EP3. My main shipping company is growing its sales or market share through introducing innovation in management	5.72	1.24	5.13		1.45	5.97		1.05	11.02**
EP4. My company applies high standards for disclosure, accounting, auditing, and social and environmental reporting	5.38	1.27	5.00		1.46	5.54		1.16	4.04
EP5. My company complies with the tax laws and regulations in all operating countries	5.67	1.06	5.32		1.42	5.81		0.84	4.78*
Productivity of the Logistics Service									
PSL1. We are happy with the level of logistics service that this provider offers us	6.02	1.11	5.71		1.42	6.15		0.92	3.56
PSL2. Improving logistics service is a high priority in our company	6.09	1.03	5.71		1.44	6.25		0.76	6.44*
PSL3. We constantly try to reduce the overall logistics cost	5.93	1.21	5.55		1.48	6.09		1.04	4.64*
PSL4. Achieving productivity through quality logistics service is critical to the success of our business	6.00	1.12	5.23		1.54	6.32		0.66	26.20**
PSL5. We are constantly trying to increase the level of global logistics service	5.97	1.02	5.42		1.36	6.20		0.74	14.46**
PSL6. The top management of the company is aware of the impact on sales of changes in the level of logistics service	5.87	1.20	5.19		1.52	6.17		0.90	17.02**
Importance of the Logistics Service									
ILS1. Logistics adds value to the relationship with the supplier and gives this supplier a competitive advantage	5.93	1.08	5.52		1.48	6.11		0.82	6.86*
ILS2. We increase orders when the level of logistics service offered is equal to or higher than our expectations	5.81	1.20	5.13		1.71	6.09		0.76	16.16**
ISL3. The top management of the company is aware of the cost implications of changes in the logistics service	5.96	1.10	5.48		1.46	6.16		0.84	8.94**
ILS4. For our clients, logistics adds value to our company and provides it with a competitive advantage	5.95	1.19	5.52		1.48	6.14		1.00	6.18*
Quantification of the Logistics Value									
QLV1. We measure and quantify the elements of the logistics service	5.85	1.08	5.35		1.49	6.05		0.77	9.92**
QLV2. We can express the value of logistics quality measurements in dollars	5.68	1.23	5.13		1.61	5.92		0.95	9.82**
Loyalty									
LO1. I will recommend the services of my main shipping company to other companies	6.13	1.10	5.65		1.49	6.33		0.82	8.53**
LO2. I will deliver positive word of mouth about the service of my main shipping company to other companies	5.97	1.14	5.58		1.50	6.14		0.90	5.16*
LO3. It is beneficial to keep the trade connection with my main shipping company	6.06	1.10	5.61		1.52	6.25		0.81	7.64*
LO4. I will extend or renew the contract with my main shipping company in the future	6.03	1.12	5.61		1.49	6.20		0.86	6.09*
LO5. I have a strong sense of loyalty to my main shipping company	5.82	1.28	5.32		1.60	6.02		1.07	6.95*

*SD* standard deviation; **p* < 0.05; ***p* < 0.01.

## References

[CR1] Alamoush AS, Ballini F, Ölçer AI (2021). Ports, maritime transport, and industry the immediate impact of COVID-19 and the way forward. Marit Technol Res.

[CR2] Anderson JC, Gerbing DW (1988). Structural equation modeling in practice: a review and recommended two-step approach. Psychol Bull.

[CR3] Arslanagic-Kalajdzic M, Zabkar V (2017). Hold me responsible: the role of corporate social responsibility and corporate reputation for client-perceived value. Corp Commun: Int J.

[CR4] Ashrafi M, Walker TR, Magnan GM, Adams M, Acciaro M (2020). A review of corporate sustainability drivers in maritime ports: a multi-stakeholder perspective. Marit Policy Manag.

[CR5] Balci G, Cetin IB, Tanyeri M (2018). Differentiation of container shipping services in Turkey. Transp Policy.

[CR6] Balci G, Caliskan A, Yuen KF (2019). Relational bonding strategies, customer satisfaction, and loyalty in the container shipping market. Int J Phys Distrib Logist Manag.

[CR7] Benamara H, Hoffmann J, Youssef F (2019). Maritime transport: the sustainability imperative.

[CR8] Chin WW (1998). The partial least squares approach to structural equation modeling. Modern Meth for Bus Res.

[CR9] Colmenares OA, Saavedra JL (2007). Theoretical review of the brand loyalty: approaches and valuations. Cuadernos De Gestión.

[CR10] CSIS (2021) El Negocio Marítimo de Panamá y el Panorama Estratégico en Evolución. https://www.csis.org/analysis/panamas-maritime-business-and-evolving-strategic-landscape. Accessed 10 December 2022

[CR11] Currás-Pérez R, Dolz-Dolz C, Miquel-Romero MJ, Sánchez-García I (2018). How social, environmental, and economic CSR affects consumer-perceived value: Does perceived consumer effectiveness make a difference?”. Corporate Social Responsib Environ Manag.

[CR12] Dike RA, Stanley CC (2021). Effect of customer relationship management on customers’loyalty in shipping companies in lagos state, Nigeria. Am Int J Bus Manag.

[CR13] Dobmeier M (2016). Understanding and managing service productivity: A literature review Journal of Business. Mark Manag.

[CR14] Elkington J, Henriques A, Richardson J (2004). Enter the triple bottom line. The triple bottom line, does it all add up?.

[CR15] Falk RF, Miller NB (1992). A primer for soft modeling.

[CR16] Fasoulis I, Kurt RE, Poutos EI (2019) A quantitative study into perceptions and attitudes of corporate social responsibility and sustainability developments in international shipping. In: 8th International Maritime Science Conference. University of Montenegro, 161–174

[CR17] Fasoulis I, Kurt RE (2019). Embracing sustainability in shipping: assessing industry’s adaptations incited by the, newly, introduced “triple bottom line” approach to sustainable maritime development. Social Sciences.

[CR18] Feo-Valero M, Martínez-Moya J (2022) Shippers vs. freight forwarders: do they differ in their port choice decisions? Evidence from the Spanish ceramic tile industry. Res Transp Econ 101195. 10.1016/j.retrec.2022.101195

[CR19] Fernando Y, Jasmi MFA, Shaharudin MS (2019). Maritime green supply chain management: its light and shadow on the bottom line dimensions of sustainable business performance. Int J Shipp Transp Logist.

[CR20] Fornell C, Larcker DF (1981). Evaluating structural equation models with unobservable variables and measurement error. J Mark Res.

[CR21] GarcíaLópez MJ (2015). La cuenta del triple resultado o triple bottom line. Revista De Contabilidad y Dirección.

[CR22] Garg CP, Kashav V (2019). Evaluating value creating factors in greening the transportation of Global Maritime Supply Chains (GMSCs) of containerized freight. Transp Res Part d: Transp Environ.

[CR23] Georgia Tech Panama Logistics Innovation and Research Center (2022). Main routes and traffic. https://logistics.gatech.pa/es/assets/panama-canal/statistics. Accessed 08 December 2022

[CR24] Gil-Saura I, Servera-Francés D, Fuentes-Blasco M (2010). Antecedents and consequences of logistics value: and empirical investigation in the Spanish market. Ind Mark Manage.

[CR25] Gil-Saura I, Berenguer-Contri G, Ruiz-Molina ME, Ospina-Pinzón S (2015). La calidad y el valor percibido en el transporte de mercancías en España y su importancia en la segmentación de clients. Innovar.

[CR26] Gil-Saura I, Berenguer-Contrí G, Ruiz-Molina E (2018). Satisfaction and loyalty in B2B relationships in the freight forwarding industry: adding perceived value and service quality into equation. Transport.

[CR27] Gil-Saura I, Ruiz-Molin ME, Berenguer-Contrí G, Seric M (2020). The role of retail equity value and relational benefits in building B2B relationships in retailing. J Relatsh Mark.

[CR28] Grönroos C, Ojasalo K (2004). Service productivity—towards a conceptualization of the transformation of inputs into economic results in services. J Bus Res.

[CR29] Gupta A, Singh RK (2020). Developing a framework for evaluating sustainability index for logistics service providers: graph theory matrix approach. Int J Product Perform Manag.

[CR30] Hair JF, Hult GTM, Ringle CM, Sarstedt M (2017). A primer on partial least squares structural equation modelling (PLS-SEM), 2ed.

[CR31] Hair JF, Risher JJ, Sarstedt M, Ringle CM (2019). When to use and how to report the results of PLS-SEM. Eur Bus Rev.

[CR32] Hanaysha JR (2018). Customer retention and the mediating role of perceived value in retail industry. World J Entrep Manag Sustain Dev.

[CR33] Hänninen N, Karjaluoto H (2017). The effect of marketing communication on business relationship loyalty. Mark Intell Plan.

[CR34] He Y, Lai KK (2014). The effect of corporate social responsibility on brand loyalty: the mediating role of brand image. Total Qual Manag Bus Excell.

[CR35] Heilig L, Lalla-Ruiz E, Voß S (2017). Digital transformation in maritime ports: analysis and a game theoretic framework. Netnomics: Econ Res Electron Networking.

[CR36] Henseler J, Ringle CM, Sarstedt M (2015). A new criterion for assessing discriminant validity in variance-based structural equation modeling. J Acad Mark Sci.

[CR37] Henseler J, Ringle CM, Sarstedt M (2016). Testing measurement invariance of composites using partial least squares. Int Mark Rev.

[CR38] Hirvonen S, Laukkanen T, Salo J (2016). Does brand orientation help B2B SMEs in gaining business growth?. J Bus Ind Mark.

[CR39] Jozef E, Kumar KM, Iranmanesh M, Foroughi B (2019). The effect of green shipping practices on multinational companies’ loyalty in Malaysia. Int J Logist Manag.

[CR40] Justavino-Castillo ME, Gil-Saura I, Fuentes-Blasco M (2020). Efectos de la sostenibilidad y del valor logístico en las relaciones entre empresas de transporte marítimo. Estudios Gerenciales.

[CR41] Khan EA, Dewan MNA, Chowdhury MMH (2016). Reflective or formative measurement model of sustainability factor? A three industry comparison. Corp Ownersh Control.

[CR42] Kim BS, Kim BY (2019). The effect of service attributes in Korean marine transportation services. J Distrib Sci.

[CR43] Kim BS, Kim BY (2020). The effect of selection factors of marine transportation service on transaction continuity. J Asian Financ Econ Bus.

[CR44] Kumar G, Goswami M (2019). Sustainable supply chain performance, its practice and impact on barriers to collaboration. Int J Product Perform Manag.

[CR45] Lam JSL (2015). Designing a sustainable maritime supply chain: A hybrid QFD-ANP approach. Transp Res Part E.

[CR46] Lin CC, Chen YJ, Wang JW (2021). Double Matching Service Preference for Promoting Short Sea Shipping. Evid Taiwan Marit Bus Rev.

[CR47] Linh NTC, Nga DQ, Trang PNT (2019). Evaluating the ability to achieve efficiency in providing services of the freight forwarding firms in Viet Nam. Int J Supply Chain Manag.

[CR48] Lun YHV, Lai KH, Wong CWY, Cheng TCE (2016). Green shipping management.

[CR49] Luo X, Bhattacharya CB (2006). Corporate social responsibility, customer satisfaction, and market value. J Mark.

[CR50] Makkonen T, Inkinen T (2021). Systems of environmental innovation: sectoral and technological perspectives on ballast water treatment systems. WMU J Marit Aff.

[CR51] MEEM (2021) Canal de Panamá. Unidad de Estadística y Administración de Modelos. https://pancanal.com/es/estadisticas/. Accessed 5 December 2022

[CR52] Mohammed A, Al-Swidi A (2019). The influence of CSR on perceived value social media and loyalty in the hotel industry. Spanish J Market – ESIC.

[CR53] Nguyen XN, Thaichon P, Nguyen Thanh PV (2019). Customer-perceived value in long-term buyer–supplier relationships: the General B2B insurance sector. Serv Mark Q.

[CR54] Novack RA, Langley CJ, Rinehart LM (1995). Creating logistics value: themes for the future.

[CR55] Oliver RL (1999). Whence consumer loyalty?. J Mark.

[CR56] Ostrowski S (2021). Commitment ladder in the relationship between service providers and customers as added value in sustainable services development. Sustainability.

[CR57] Pang K, Lu CS (2018). Organizational motivation, employee job satisfaction and organizational performance: An empirical study of container shipping companies in Taiwan. Marit Bus Rev.

[CR58] Parviainen T, Lehikoinen A, Kuikka S, Haapasaari P (2018). How can stakeholders promote environmental and social responsibility in the shipping industry?. WMU J Marit Aff.

[CR59] Psaraftis HN (2019). Sustainable shipping: a cross-disciplinary view. Marit Econ Logist.

[CR60] Ren J, Lützen M (2017). Selection of sustainable alternative energy source for shipping: multi-criteria decision making under incomplete information. Renew Sustain Energy Rev.

[CR61] Ruiz-Martínez A, Frasquet M, Gil-Saura I (2019). How to measure B2B relationship value to increase satisfaction and loyalty. J Bus Ind Mark.

[CR62] Sarstedt M, Henseler J, Ringle CM (2011). Multigroup analysis in partial least squares (PLS) path modeling: Alternative methods and empirical results. Adv Int Mark.

[CR63] Seddiek BS, Ammar NR (2020). Harnessing wind energy on merchant ships: case study Flettnerrotors onboard bulk carriers. Environ Sci Pollut Res.

[CR64] Servera-Francés D, Gil-Saura I, Fuentes-Blasco M (2008). El valor logístico: una propuesta de modelo a partir de sus antecedentes y consecuencias. Revista Europea De Dirección y Economía De La Empresa.

[CR65] Servera-Francés D, Fuentes-Blasco M, Piqueras-Tomás L (2020). The importance of sustainable practices in value creation and consumers’ commitment with companies’ commercial format. Sustainability.

[CR66] Shin Y, Thai VV, Grewal D, Kim Y (2017). Do corporate sustainable management activities improve customer satisfaction, word of mouth intention and repurchase intention? Empirical evidence from the shipping industry. Int J Logist Manag.

[CR67] Spychalska-Wojtkiewicz M (2020). The relation between sustainable development trends and customer value management. Sustainability.

[CR68] Tepe R, Arabelen G (2022). Relationship marketing strategies in the container shipping industry: A qualitative approach. Sci J Zeszyty Naukowe Marit Univ Szczecin.

[CR69] Tran TMT, Yuen KF, Wang X, Li KX (2020). The antecedents of sustainable shipping management and organisational performance: resource accumulation and orientation perspectives. Int J Phys Distrib Logist Manag.

[CR70] UNCTAD (2020) Review of maritime transport 2020. https://unctad.org/system/files/official-document/rmt2020_en.pdf. Accessed 26 November 2022

[CR71] UNCTAD (2021) Review of maritime transport 2021. https://unctad.org/system/files/official-document/rmt2021summary_en.pdf. Accessed 26 November 2022

[CR72] UNCTAD (2022) Transporte sostenible y resiliente y facilitación del comercio en tiempos de pandemia y más allá: principales retos y oportunidades. https://unctad.org/system/files/official-document/cimem7d26_es.pdf. Accessed 21 November 2022

[CR73] Van-den-Berg R, de Langen PW (2015). Assessing the intermodal value proposition of shipping lines: Attitudes of shippers and forwarders. Marit Econ Logist.

[CR74] Van-den-Berg R, de Langen PW (2017). Environmental sustainability in container transport: the attitudes of shippers and forwarders. Int J Log Res Appl.

[CR75] Vejvar M, Lai KH, Lo C, Fürst E (2018). Strategic responses to institutional forces pressuring sustainability practice adoption: Case-based evidence from inland port operations. Transp Res Part d: Transp Environ.

[CR76] Vural CA, Göçer A, Halldorsson A (2019). Value co-creation in maritime logistics networks: A service triad perspective. Transp Policy.

[CR77] Vural CA, Baştuğ S, Gülmez S (2021). Sustainable brand positioning by container shipping firms Evidence from social media communications. Transp Res Part D: Transp Environ.

[CR78] Yang CS (2017). An analysis of institutional pressures, green supply chain management, and green performance in the container shipping context. Transp Res Part d: Transp Environ.

[CR79] Yazdanparast A, Manuj I, Swartz SM (2010). Co-creating logistics value: a service-dominant logic perspective. Int J Logist Manag.

[CR80] Yuan CL, Moon H, Kim KH, Wang S, Yu X (2020). Third-party organization endorsement impacts on perceived value and B2B customer loyalty. Ind Mark Manage.

[CR81] Yuen KF, Thai VV (2015). Service quality and customer satisfaction in liner shipping. Int J Qual Serv Sci.

[CR82] Yuen KF, Thai VV, Wong YD (2016). The effect of continuous improvement capacity on the relationship between of corporate social performance and business performance in maritime transport in Singapore. Transp Res Part e: Logist Transp Rev.

[CR83] Yuen KF, Thai VV, Wong YD (2016). Are customers willing to pay for corporate social responsibility? A study of individual-specific mediators. Total Qual Manag Bus Excell.

[CR84] Yuen KF, Wang X, Wong YD, Zhou Q (2017). Antecedents and outcomes of sustainable shipping practices: The integration of stakeholder and behavioural theories. Transp Res Part e: Logist Transp Rev.

[CR85] Yuen KF, Thai VV, Wong YD (2018). An investigation of shippers’ satisfaction and behaviour towards corporate social responsibility in maritime transport. Transp Res Part a: Pol Practice.

[CR86] Yuen KF, Wang X, Wong YD, Ma F (2019). A contingency view of the effects of sustainable shipping exploitation and exploration on business performance. Transp Policy.

[CR87] Yuen KF, Wang X, Wong, YD, Zhou Q (2018b) The effect of sustainable shipping practices on shippers’ loyalty: the mediating role of perceived value, trust and transaction cost. Transportation Research Part E: Logistics and Transportation Review 116 (123–135). 10.1016/j.tre.2018b.06.002

[CR88] Zhou Y, Wang X, Yuen KF (2021). Sustainability disclosure for container shipping: a text-mining approach. Transp Policy.

